# Supplementary material for: Genome-Wide mRNA and Long Non-Coding RNA Analysis of Porcine Trophoblast Cells Infected with Porcine Reproductive and Respiratory Syndrome Virus Associated with Reproductive Failure
Source: Int J Mol Sci. 2023 Jan 4;24(2):919. doi: 10.3390/ijms24020919 (PMC9866684; doi:10.3390/ijms24020919)
Supplement: Supplementary file 1 [file ijms-24-00919-s001.zip › Supplement Table S1.pdf]

Supplement Table S1:

The top 20 KEGG pathways of dysfunctional mRNAs

Pathway term	Rich factor	Q value	Gene number
Chronic myeloid leukemia	0.536231884	0.21491223	37
Prostate cancer	0.505882353	0.21491223	43
Notch signaling pathway	0.590909091	0.21491223	26
Acute myeloid leukemia	0.555555556	0.21491223	30
Thyroid hormone signaling pathway	0.451327434	0.227748452	51
Protein processing in endoplasmic reticulum	0.413580247	0.227748452	67
Epstein-Barr virus infection	0.402116402	0.227748452	76
Ubiquitin mediated proteolysis	0.425373134	0.227748452	57
mRNA surveillance pathway	0.464285714	0.227748452	39
Hippo signaling pathway	0.420289855	0.227748452	58
Pathways in cancer	0.369905956	0.227748452	118
Lysine degradation	0.52	0.24312213	26
Cell cycle	0.426086957	0.252596335	49
AMPK signaling pathway	0.423728814	0.252596335	50
Adherens junction	0.464788732	0.263236493	33
Endocytosis	0.383495146	0.263236493	79
Hepatitis B	0.397350993	0.3036949	60
Spliceosome	0.403100775	0.327198397	52
Endometrial cancer	0.489795918	0.327198397	24
Bladder cancer	0.514285714	0.415456227	18
