# Supplementary material for: Genome-Wide mRNA and Long Non-Coding RNA Analysis of Porcine Trophoblast Cells Infected with Porcine Reproductive and Respiratory Syndrome Virus Associated with Reproductive Failure
Source: Int J Mol Sci. 2023 Jan 4;24(2):919. doi: 10.3390/ijms24020919 (PMC9866684; doi:10.3390/ijms24020919)
Supplement: Supplementary file 1 [file ijms-24-00919-s001.zip › Supplement Table S2.pdf]

Supplement Table S2:

The top 20 KEGG pathways of dysfunctional lncRNAs

Pathway term	Rich factor	Q value	Gene number
ABC transporters	0.209302	0.089842	9
Amoebiasis	0.140187	0.089842	15
Arrhythmogenic right ventricular cardiomyopathy (ARVC)	0.149254	0.173916	10
Propanoate metabolism	0.230769	0.173916	6
Protein digestion and absorption	0.1375	0.173916	11
Bile secretion	0.144928	0.173916	10
Focal adhesion	0.097938	0.242681	19
Vasopressin-regulated water reabsorption	0.162791	0.276446	7
Fatty acid metabolism	0.155556	0.299105	7
ECM-receptor interaction	0.121951	0.299105	10
Pancreatic secretion	0.114943	0.383213	10
Axon guidance	0.10084	0.466818	12
Estrogen signaling pathway	0.103093	0.502549	10
Nicotine addiction	0.151515	0.502549	5
Dilated cardiomyopathy	0.107143	0.502549	9
Dorso-ventral axis formation	0.173913	0.502549	4
Fatty acid biosynthesis	0.230769	0.502549	3
Insulin secretion	0.104651	0.502549	9
PI3K-Akt signaling pathway	0.073171	0.674516	24
cAMP signaling pathway	0.079602	0.69516	16
